# 4,4′-Dibromo-2,2′-[*m*-phenyl­enebis(nitrilo­methanylyl­idene)]diphenol

**DOI:** 10.1107/S1600536811030431

**Published:** 2011-08-02

**Authors:** Kwang Ha

**Affiliations:** a School of Applied Chemical Engineering, The Research Institute of Catalysis, Chonnam National University, Gwangju 500-757, Republic of Korea

## Abstract

The title compound, C_20_H_14_Br_2_N_2_O_2_, is a dibasic tetra­dentate Schiff base and reveals intra­molecular O—H⋯N hydrogen bonds between the hy­droxy O atoms and the imino N atoms. The dihedral angle between the central and terminal benzene rings is 39.7 (1)°. In the crystal, the compound is disposed about a crystallographic mirror plane parallel to the *ac* plane passing through the two central C atoms. The mol­ecules are stacked in columns along the *c* axis through π–π inter­actions, the shortest centroid–centroid distance being 3.872 (3) Å.

## Related literature

For the crystal structure of 4,4′-dibromo-2,2′-[1,2-phenyl­enebis(nitrilo­methanylyl­idene)]diphenol, see: Kabak *et al.* (2000 ▶).
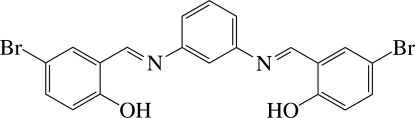


## Experimental

### 

#### Crystal data

C_20_H_14_Br_2_N_2_O_2_
*M* *_r_* = 474.15
Orthorhombic, 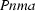

*a* = 12.326 (2) Å
*b* = 37.226 (6) Å
*c* = 3.8726 (7) Å
*V* = 1776.9 (5) Å^3^
*Z* = 4
Mo *K*α radiation
μ = 4.58 mm^−1^
*T* = 200 K
0.21 × 0.08 × 0.06 mm

#### Data collection

Bruker SMART 1000 CCD diffractometer
Absorption correction: multi-scan (*SADABS*; Bruker, 2000 ▶) *T*_min_ = 0.578, *T*_max_ = 0.760
11852 measured reflections
2236 independent reflections
1332 reflections with *I* > 2σ(*I*)
*R*_int_ = 0.093

#### Refinement

*R*[*F* ^2^ > 2σ(*F* ^2^)] = 0.046
*wR*(*F* ^2^) = 0.100
*S* = 1.03
2236 reflections
125 parameters
H atoms treated by a mixture of independent and constrained refinement
Δρ_max_ = 1.02 e Å^−3^
Δρ_min_ = −0.62 e Å^−3^

### 

Data collection: *SMART* (Bruker, 2000 ▶); cell refinement: *SAINT* (Bruker, 2000 ▶); data reduction: *SAINT*; program(s) used to solve structure: *SHELXS97* (Sheldrick, 2008 ▶); program(s) used to refine structure: *SHELXL97* (Sheldrick, 2008 ▶); molecular graphics: *ORTEP-3* (Farrugia, 1997 ▶) and *PLATON* (Spek, 2009 ▶); software used to prepare material for publication: *SHELXL97*.

## Supplementary Material

Crystal structure: contains datablock(s) global, I. DOI: 10.1107/S1600536811030431/is2759sup1.cif

Structure factors: contains datablock(s) I. DOI: 10.1107/S1600536811030431/is2759Isup2.hkl

Additional supplementary materials:  crystallographic information; 3D view; checkCIF report

## Figures and Tables

**Table 1:** Hydrogen-bond geometry (Å, °)

*D*—H⋯*A*	*D*—H	H⋯*A*	*D*⋯*A*	*D*—H⋯*A*
O1—H1⋯N1	0.82 (4)	1.88 (4)	2.617 (5)	150 (5)

**Fig. 1.**
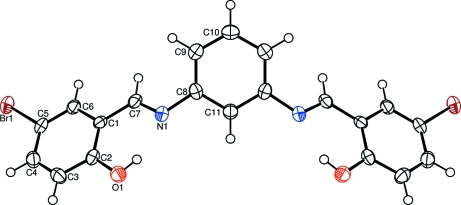
The structure of the title compound, with displacement ellipsoids drawn at the 50% probability level; H atoms are shown as small circles of arbitrary radius. Unlabelled atoms are related to the reference atoms by the (x, 1/2 - y, z) symmetry transformation.

**Fig. 2.**
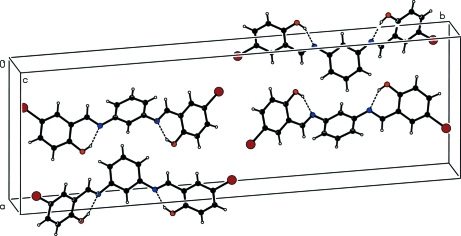
View of the unit-cell contents of the title compound. Hydrogen-bond interactions are drawn with dashed lines.

## supplementary crystallographic information

### Comment

The title compound, C_20_H_14_Br_2_N_2_O_2_, is a tetradentate Schiff base (Fig. 1), which can act as a dibasic ligand, *i.e.* the N and O donor atoms can coordinate one or two metal ions. The compound crystallized in the orthorhombic space group *Pnma*, whereas the analogous Schiff base with 1,2-phenylene group crystallized in the different orthorhombic space group *Pbca* (Kabak *et al.*, 2000).

The compound is disposed about a crystallographic mirror plane parallel to the *ac* plane passing through the two central C atoms (C10 and C11) at the special positions (*x*, 1/4, *z*; Wyckoff letter c). In the crystal structure, the three benzene rings are not parallel: the dihedral angle between the central benzene ring and the lateral benzene ring is 39.7 (1)°, and the dihedral angle between the lateral benzene rings is 41.7 (1)°. The Schiff base reveals strong intramolecular O—H···N hydrogen bonding between the hydroxy O atom and the imino N atom with *d*(O···N) = 2.617 (5) Å forming a nearly planar six-membered ring (Fig. 2, Table 1). The N1—C7/8 bond lengths and the C7—N1—C8 bond angle indicate that the imino N1 atom is *sp*^2^-hybridized [*d*(N1═C7) = 1.287 (5) Å and *d*(N1—C8) = 1.438 (5) Å; <C7—N1—C8 = 118.3 (4)°]. The molecules are stacked in columns along the *c* axis. When viewed down the *b* axis, the successive compounds are stacked in the opposite direction. In the columns, π–π interactions between benzene rings are present, the shortest centroid-centroid distance being 3.872 (3) Å, and the ring planes are parallel and shifted for 1.461 Å.

### Experimental

1,3-Phenylenediamine (0.7567 g, 6.997 mmol) and 5-bromosalicylaldehyde (2.8150 g, 14.004 mmol) in EtOH (30 ml) were stirred for 2 h at room temperature. After addition of pentane (30 ml) to the reaction mixture, the formed precipitate was separated by filtration, washed with ether, and dried at 50 °C, to give a yellow powder (3.0997 g). Crystals suitable for X-ray analysis were obtained by slow evaporation from an ethylacetate solution.

### Refinement

H atoms were positioned geometrically and allowed to ride on their respective parent atoms [C—H = 0.95 Å and *U*_iso_(H) = 1.2*U*_eq_(C)]. The hydroxy H atom was located in a Fourier difference map and refined isotropically [O—H = 0.82 (4) Å].

### Crystal data

**Table:**

C_20_H_14_Br_2_N_2_O_2_	*F*(000) = 936
*M**_r_* = 474.15	*D*_x_ = 1.772 Mg m^−^^3^
Orthorhombic, *P**n**m**a*	Mo *K*α radiation, λ = 0.71073 Å
Hall symbol: -P 2ac 2n	Cell parameters from 2519 reflections
*a* = 12.326 (2) Å	θ = 2.2–26.1°
*b* = 37.226 (6) Å	µ = 4.58 mm^−^^1^
*c* = 3.8726 (7) Å	*T* = 200 K
*V* = 1776.9 (5) Å^3^	Stick, yellow
*Z* = 4	0.21 × 0.08 × 0.06 mm

### Data collection

**Table:**

Bruker SMART 1000 CCD diffractometer	2236 independent reflections
Radiation source: fine-focus sealed tube	1332 reflections with *I* > 2σ(*I*)
graphite	*R*_int_ = 0.093
φ and ω scans	θ_max_ = 28.3°, θ_min_ = 2.2°
Absorption correction: multi-scan (*SADABS*; Bruker, 2000)	*h* = −16→16
*T*_min_ = 0.578, *T*_max_ = 0.760	*k* = −40→49
11852 measured reflections	*l* = −5→5

### Refinement

**Table:**

Refinement on *F*^2^	Primary atom site location: structure-invariant direct methods
Least-squares matrix: full	Secondary atom site location: difference Fourier map
*R*[*F*^2^ > 2σ(*F*^2^)] = 0.046	Hydrogen site location: inferred from neighbouring sites
*wR*(*F*^2^) = 0.100	H atoms treated by a mixture of independent and constrained refinement
*S* = 1.03	*w* = 1/[σ^2^(*F*_o_^2^) + (0.0335*P*)^2^] where *P* = (*F*_o_^2^ + 2*F*_c_^2^)/3
2236 reflections	(Δ/σ)_max_ < 0.001
125 parameters	Δρ_max_ = 1.02 e Å^−^^3^
0 restraints	Δρ_min_ = −0.62 e Å^−^^3^

### Special details

**Table:**

Geometry. All e.s.d.'s (except the e.s.d. in the dihedral angle between two l.s. planes) are estimated using the full covariance matrix. The cell e.s.d.'s are taken into account individually in the estimation of e.s.d.'s in distances, angles and torsion angles; correlations between e.s.d.'s in cell parameters are only used when they are defined by crystal symmetry. An approximate (isotropic) treatment of cell e.s.d.'s is used for estimating e.s.d.'s involving l.s. planes.
Refinement. Refinement of *F*^2^ against ALL reflections. The weighted *R*-factor *wR* and goodness of fit *S* are based on *F*^2^, conventional *R*-factors *R* are based on *F*, with *F* set to zero for negative *F*^2^. The threshold expression of *F*^2^ > σ(*F*^2^) is used only for calculating *R*-factors(gt) *etc*. and is not relevant to the choice of reflections for refinement. *R*-factors based on *F*^2^ are statistically about twice as large as those based on *F*, and *R*- factors based on ALL data will be even larger.

### Fractional atomic coordinates and isotropic or equivalent isotropic displacement parameters (Å^2^)

**Table:**

	*x*	*y*	*z*	*U*_iso_*/*U*_eq_	
Br1	0.34154 (4)	0.027860 (11)	0.17780 (12)	0.03517 (17)	
O1	0.6141 (3)	0.14597 (9)	0.8028 (10)	0.0411 (9)	
H1	0.575 (4)	0.1631 (12)	0.844 (12)	0.040 (16)*	
N1	0.4386 (3)	0.18548 (9)	0.8276 (9)	0.0290 (8)	
C1	0.4402 (3)	0.12540 (10)	0.6019 (11)	0.0258 (10)	
C2	0.5508 (4)	0.12003 (10)	0.6591 (11)	0.0272 (10)	
C3	0.5980 (4)	0.08728 (12)	0.5711 (12)	0.0352 (12)	
H3	0.6731	0.0834	0.6119	0.042*	
C4	0.5359 (4)	0.06033 (11)	0.4244 (12)	0.0340 (11)	
H4	0.5686	0.0381	0.3621	0.041*	
C5	0.4268 (4)	0.06563 (11)	0.3688 (11)	0.0280 (10)	
C6	0.3776 (3)	0.09764 (10)	0.4542 (11)	0.0273 (10)	
H6	0.3022	0.1010	0.4141	0.033*	
C7	0.3858 (4)	0.15904 (11)	0.6948 (11)	0.0289 (10)	
H7	0.3100	0.1614	0.6563	0.035*	
C8	0.3799 (4)	0.21767 (10)	0.9126 (11)	0.0269 (10)	
C9	0.2764 (4)	0.21769 (11)	1.0496 (12)	0.0306 (10)	
H9	0.2403	0.1957	1.0956	0.037*	
C10	0.2259 (5)	0.2500	1.1193 (16)	0.0323 (15)	
H10	0.1552	0.2500	1.2168	0.039*	
C11	0.4341 (5)	0.2500	0.8520 (16)	0.0286 (14)	
H11	0.5068	0.2500	0.7706	0.034*	

### Atomic displacement parameters (Å^2^)

**Table:**

	*U*^11^	*U*^22^	*U*^33^	*U*^12^	*U*^13^	*U*^23^
Br1	0.0442 (3)	0.0248 (3)	0.0364 (3)	−0.0065 (2)	−0.0016 (2)	−0.0010 (2)
O1	0.0263 (19)	0.0294 (19)	0.068 (3)	−0.0016 (15)	−0.0048 (17)	−0.0060 (18)
N1	0.030 (2)	0.0231 (19)	0.034 (2)	−0.0012 (15)	0.0010 (18)	0.0022 (17)
C1	0.020 (2)	0.024 (2)	0.033 (3)	−0.0036 (17)	0.0010 (19)	0.0052 (18)
C2	0.032 (3)	0.024 (2)	0.026 (2)	−0.0044 (18)	0.001 (2)	0.0041 (19)
C3	0.029 (3)	0.031 (3)	0.046 (3)	0.0060 (19)	−0.003 (2)	0.000 (2)
C4	0.040 (3)	0.026 (3)	0.037 (3)	0.003 (2)	0.004 (2)	0.003 (2)
C5	0.033 (3)	0.023 (2)	0.028 (3)	−0.0092 (18)	−0.001 (2)	0.0034 (18)
C6	0.026 (2)	0.026 (2)	0.030 (3)	−0.0030 (18)	−0.002 (2)	0.0063 (19)
C7	0.027 (3)	0.026 (2)	0.034 (3)	−0.0034 (18)	0.003 (2)	0.008 (2)
C8	0.030 (3)	0.023 (2)	0.027 (3)	0.0033 (18)	−0.005 (2)	−0.0015 (18)
C9	0.031 (3)	0.029 (2)	0.031 (3)	−0.0040 (19)	−0.001 (2)	0.002 (2)
C10	0.029 (4)	0.039 (4)	0.029 (4)	0.000	0.003 (3)	0.000
C11	0.026 (4)	0.027 (3)	0.033 (4)	0.000	−0.004 (3)	0.000

### Geometric parameters (Å, °)

**Table:**

Br1—C5	1.905 (4)	C4—H4	0.9500
O1—C2	1.361 (5)	C5—C6	1.378 (5)
O1—H1	0.82 (4)	C6—H6	0.9500
N1—C7	1.287 (5)	C7—H7	0.9500
N1—C8	1.438 (5)	C8—C9	1.381 (6)
C1—C2	1.395 (6)	C8—C11	1.396 (5)
C1—C6	1.411 (5)	C9—C10	1.381 (5)
C1—C7	1.465 (6)	C9—H9	0.9500
C2—C3	1.394 (6)	C10—C9^i^	1.381 (5)
C3—C4	1.385 (6)	C10—H10	0.9500
C3—H3	0.9500	C11—C8^i^	1.396 (5)
C4—C5	1.375 (6)	C11—H11	0.9500
			
C2—O1—H1	107 (3)	C5—C6—H6	120.3
C7—N1—C8	118.3 (4)	C1—C6—H6	120.3
C2—C1—C6	119.6 (4)	N1—C7—C1	121.3 (4)
C2—C1—C7	122.0 (4)	N1—C7—H7	119.3
C6—C1—C7	118.4 (4)	C1—C7—H7	119.3
O1—C2—C3	118.8 (4)	C9—C8—C11	120.4 (4)
O1—C2—C1	121.6 (4)	C9—C8—N1	123.6 (4)
C3—C2—C1	119.7 (4)	C11—C8—N1	116.0 (4)
C4—C3—C2	120.2 (4)	C10—C9—C8	119.5 (4)
C4—C3—H3	119.9	C10—C9—H9	120.3
C2—C3—H3	119.9	C8—C9—H9	120.3
C5—C4—C3	120.1 (4)	C9—C10—C9^i^	121.1 (6)
C5—C4—H4	120.0	C9—C10—H10	119.4
C3—C4—H4	120.0	C9^i^—C10—H10	119.4
C4—C5—C6	121.1 (4)	C8—C11—C8^i^	119.1 (6)
C4—C5—Br1	119.6 (3)	C8—C11—H11	120.5
C6—C5—Br1	119.2 (3)	C8^i^—C11—H11	120.5
C5—C6—C1	119.3 (4)		
			
C6—C1—C2—O1	−179.6 (4)	C7—C1—C6—C5	−179.2 (4)
C7—C1—C2—O1	−0.4 (6)	C8—N1—C7—C1	−180.0 (4)
C6—C1—C2—C3	−0.3 (6)	C2—C1—C7—N1	1.4 (6)
C7—C1—C2—C3	178.9 (4)	C6—C1—C7—N1	−179.4 (4)
O1—C2—C3—C4	180.0 (4)	C7—N1—C8—C9	38.4 (6)
C1—C2—C3—C4	0.7 (7)	C7—N1—C8—C11	−142.3 (5)
C2—C3—C4—C5	−0.8 (7)	C11—C8—C9—C10	1.4 (7)
C3—C4—C5—C6	0.5 (7)	N1—C8—C9—C10	−179.2 (4)
C3—C4—C5—Br1	−178.4 (3)	C8—C9—C10—C9^i^	0.9 (9)
C4—C5—C6—C1	−0.1 (6)	C9—C8—C11—C8^i^	−3.8 (8)
Br1—C5—C6—C1	178.8 (3)	N1—C8—C11—C8^i^	176.9 (3)
C2—C1—C6—C5	0.0 (6)		

Symmetry codes: (i) *x*, −*y*+1/2, *z*.

### Hydrogen-bond geometry (Å, °)

**Table:**

*D*—H···*A*	*D*—H	H···*A*	*D*···*A*	*D*—H···*A*
O1—H1···N1	0.82 (4)	1.88 (4)	2.617 (5)	150 (5)
